# Adequately increased postoperative medial proximal tibial angle can prevent medial joint space narrowing after open-wedge high tibial osteotomy: a 3-year cohort study

**DOI:** 10.1186/s43019-026-00338-0

**Published:** 2026-10-29

**Authors:** Daisuke Chiba, Shugo Maeda, Eiji Sasaki, Takahiro Tsushima, Yuka Kimura, Eiichi Tsuda, Yasuyuki Ishibashi

**Affiliations:** 1 Department of Orthopaedic Surgery, Hirosaki University Graduate School of Medicine, Hirosaki, Japan; 2 Department of Orthopaedic Surgery, Aomori Rosai Hospital, Hachinohe, Japan; 3 Department of Rehabilitation Medicine, Hirosaki University Graduate School of Medicine, Hirosaki, Japan

**Keywords:** Knee osteoarthritis, Medial open-wedge high tibial osteotomy, Medial proximal tibial angle, Joint space narrowing, Osteophyte formation

## Abstract

**Purpose:**

This study aimed to investigate the longitudinal relationship between postoperative medial proximal tibial angle (pMPTA) and joint space narrowing or osteophyte formation during a 3-year follow-up period after medial open-wedge high tibial osteotomy (MOWHTO).

**Methods:**

This retrospective cohort study included 133 knees of 101 patients (58 female patients; 77 female knees) who underwent MOWHTO [mean age: 57.5 ± 7.9 years; body mass index: 26.3 ± 4.0 kg/m^2^; 32 patients (19 female patients) underwent bilateral surgeries]. On the basis of plain radiograph until 1 month postoperatively (Post Op), pMPTA and change in MPTA (ΔMPTA) (i.e., [pMPTA] − [preoperative MPTA]) were measured. Additionally, the medial and lateral femorotibial joint space widths (MJSW/LJSW) and osteophyte area (MOPA/LOPA) were measured at Post Op, and the JSW and OPA were remeasured at 3-year follow-up (3Y). Amount of changes in JSW and OPA were measured using the following formula: Δ = [JSW_3Y_ or OPA_3Y_] − [JSW_Post Op_ or OPA_Post Op_]. If ΔMJSW was ≤ −0.3 mm, we defined medial knee osteoarthritis as progressed (p-mOA).

**Results:**

Forty-seven (35.3%) patients were diagnosed with p-mOA. Both pMPTA and ΔMPTA positively correlated with ΔMJSW. Receiver operating characteristic curves of pMPTA detected p-mOA, and the cut-off of pMPTA was 90°. Generalized estimation equations revealed that those with < 90° of correction for pMPTA demonstrated higher odds ratio of p-mOA (odds ratio: 3.300; 95% confidence interval: 1.327–8.198, *p*-value: 0.010), as compared with those with ≥ 90° correction. This finding was more robust in patients with preoperative Kellgren–Lawrence grade 0 or 1 (odds ratio: 5.408; 95% confidence interval: 1.400–20.905, *p*-value: 0.014), and ΔLJSW did not correlate with both pMPTA and ΔMPTA. Both the MOPA and LOPA increased during the 3-year follow-up period. However, there was no correlation between ΔOPA and pMPTA or ΔMPTA.

**Conclusions:**

Only in those with nonradiographic OA, correcting pMPTA with < 90° demonstrated a higher odds ratio of medial femorotibial joint space narrowing after MOWHTO when compared with those having ≥ 90° correction in the current short-term follow-up.

*Level of evidence*: III.

**Supplementary Information:**

The online version contains supplementary material available at https://doi.org/10.1186/s43019-026-00338-0.

## Introduction

Medial open-wedge high tibial osteotomy (MOWHTO) is an effective surgical treatment for active patients with medial compartment osteoarthritis (OA) with varus alignment [1–3]. MOWHTO is an alignment correction procedure that alters the load from an involved medial compartment to a relatively healthy lateral compartment by shifting the mechanical axis (MA) from medial to lateral [2, 4, 5]. In a previous biomechanical study to clarify how frontal varus deformity affected the loading pattern of cadaveric knees, greater contact pressure was observed when an axial load of 1000 N was applied to the medial compartment. By contrast, this relationship was reversed for a valgus MA of 62%, and a lateral shift of the MA caused a higher contact pressure on the lateral compartment and lower contact pressure on the medial compartment [2]. Therefore, MOWHTO is beneficial for patients with medial unicompartmental OA because it reduces mechanical stress on the inner side of the knee joint and improves the biomechanical environment in the medial compartment [6–8]. Clinically, MOWHTO improves outcomes and delays the need for joint replacement surgery by redistributing the load applied to the knee joint [9–12].

In particular, MOWHTO is commonly associated with an increase in postoperative medial proximal tibial angle (MPTA; angle between the MA of the tibia and articular surface line of the medial proximal tibia) [13] MPTA was utilized as the preferred choice of static measurement over other angles because it is independent of OA grade, single-leg/double-leg standing position, and stance width during radiographic filming [14]. Lowering MPTA to the normal range (85–90°) can be challenging, and a relatively larger postoperative MPTA (pMPTA) may be planned to achieve the targeted mechanical alignment and prevent under-correction during MOWHTO [15]. By contrast, a larger pMPTA can have negative effects on gait pattern and knee kinematics such as eccentric valgus shear stress on the articular cartilage of the lateral compartment [16, 17]. Despite the clinical relevance of MPTA, little evidence is available regarding the association between the series of MPTA during MOWHTO and radiographic changes in both medial and lateral compartments in terms of joint space width (JSW) [15, 18] and osteophytes. Therefore, this study aimed to clarify the longitudinal relationship between the series of MPTA during MOWHTO and radiographic osteoarthritic changes, such as joint space narrowing and osteophyte formation, during a 3-year follow-up period. This 3-year cohort study has two hypotheses. First, a lower MPTA is not sufficient to prevent osteoarthritic changes in the medial femorotibial compartment. Second, a greater MPTA worsens the lateral femorotibial compartment.

## Materials and methods

**Fig. 1:**
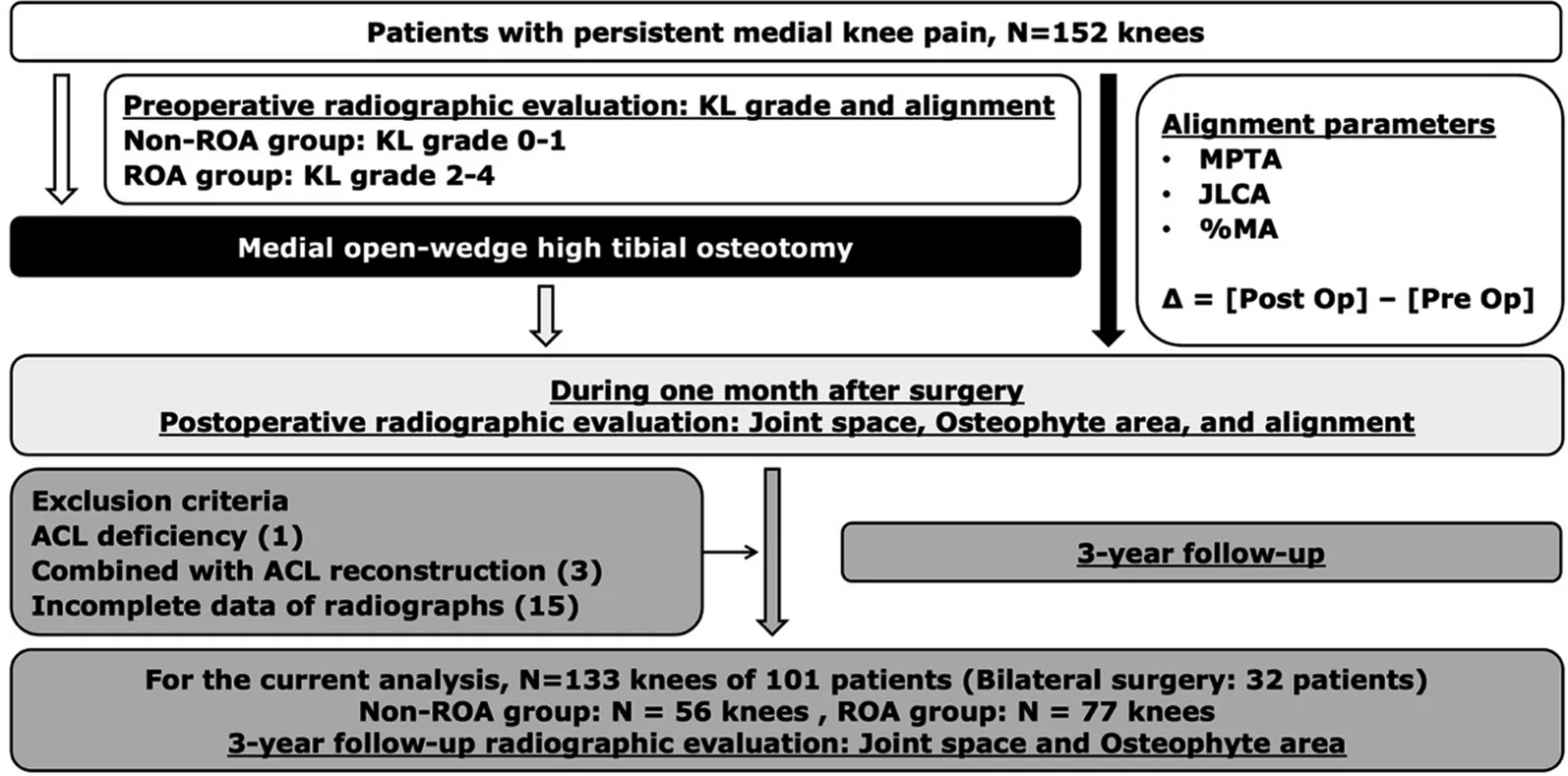
Flowchart to explain the recruitment of the current study’s subjects. *ACL* Anterior cruciate ligament, *JLCA* joint line convergence angle, *MPTA* medial proximal tibial angle, *Post Op* postoperative alignment parameters, *Pre Op* preoperative alignment parameters, *ROA* radiographic knee osteoarthritis (Kellgren–Lawrence grade ≥ 2), *%MA* % mechanical axis

From March 2006 to July 2020, this retrospective cohort study recruited 152 knees that were performed by MOWHTO for medial unicompartmental knee OA by multiple surgeons at our institution. As for the inclusion criteria, our surgical indication for MOWHTO is for patients who have medial femorotibial joint OA with consistent medial knee pain during daily activities, despite conservative treatment, having difficulty in improving their knee pain. All participants did not have flexion contracture greater than 15°. Diagnosis was made on the basis of plain standing radiographs and magnetic resonance imaging (MRI) of the involved knee joint. According to weight-bearing full-length lower extremity radiographs, all the patients demonstrated varus alignment, with their %MA passing through < 40% of the width of the tibial plateau in relation to the medial end. After reviewing medical record, we excluded the patients having anterior cruciate ligament (ACL) insufficiency (*n* = 1) and MOWHTO with ACL reconstruction (*n* = 3) at the postoperative period, and the incomplete data of radiographs (*n* = 15) during 3-year follow-up. Finally, total of 133 knees of 101 patients (58 female patients; 77 female knees) who underwent MOWHTO were included in this study [mean age: 57.5 ± 7.9 years; body mass index (BMI): 26.3 ± 4.0 kg/m^2^; 32 patients (19 female patients) underwent bilateral surgery, Fig. 1]. The ethical committee of our hospital provided permission to conduct the current study (IRB No. 2019-1096-1). Before we performed MOWHTO, all the participants had been informed for us to analyze clinical and radiographic data, and they had consented to participate in this retrospective cohort study.

### Surgical technique of open-wedge high tibial osteotomy

After arthroscopy, a longitudinal skin incision was made on the medial side of the proximal tibia. Along the superior border of the pes anserinus, the first layer was incised and the pes anserinus was retracted posteriorly to expose the superficial medial collateral ligament (MCL). After determining the osteotomy level using fluoroscopy, the superficial MCL was transected using a chisel and scalpel. On the basis of a fluoroscopic imaging, monoplane or biplane oblique osteotomy was performed, leaving a 10-mm width of an intact lateral tibial cancellous bone as a hinge to open the medial aspect of the osteotomy [19]. Thereafter, we gradually opened the osteotomy site by referencing the alignment rod to connect the hip and ankle joint centers. We controlled the MA to pass through the slope of the lateral tibial plateau, which corresponded to 62.5% of the width of the tibial plateau relative to the medial end [20]. To maintain the determined opening gap, a wedged β-tricalcium phosphate block (HOYA Technosurgical Co., Ltd.) was inserted into the posterior lesion of the opening. Finally, the proximal tibia was fixed by various plate systems: Puddu plate (Arthrex®), Position HTO plate (Aesculap®), and Tris Medial HTO plate system (OLYMPUS®). If there was an unstable medial meniscal tear involving the red–red or red–white zone, we performed arthroscopic meniscus repair, including the pull-out technique for the posterior root tear. By contrast, there was an unstable medial meniscal tear with severe degeneration involving the white–white zone, and we conducted partial meniscal resection to preserve meniscal function as much as possible. Regarding postoperative rehabilitation, 3 weeks of nonweight bearing using crutches was performed for the patients who underwent MOWHTO in cases fixed by the Puddu or position HTO plate. Thereafter, gradual weightbearing was allowed in accordance with the patient’s knee pain. Patients who underwent MOWHTO using the Tris plate system were allowed to perform weight-bearing exercises a day after the operation. Range of motion exercises were allowed on postoperative day one.

### Measurement of radiographic parameters for knee OA and alignment

**Fig. 2:**
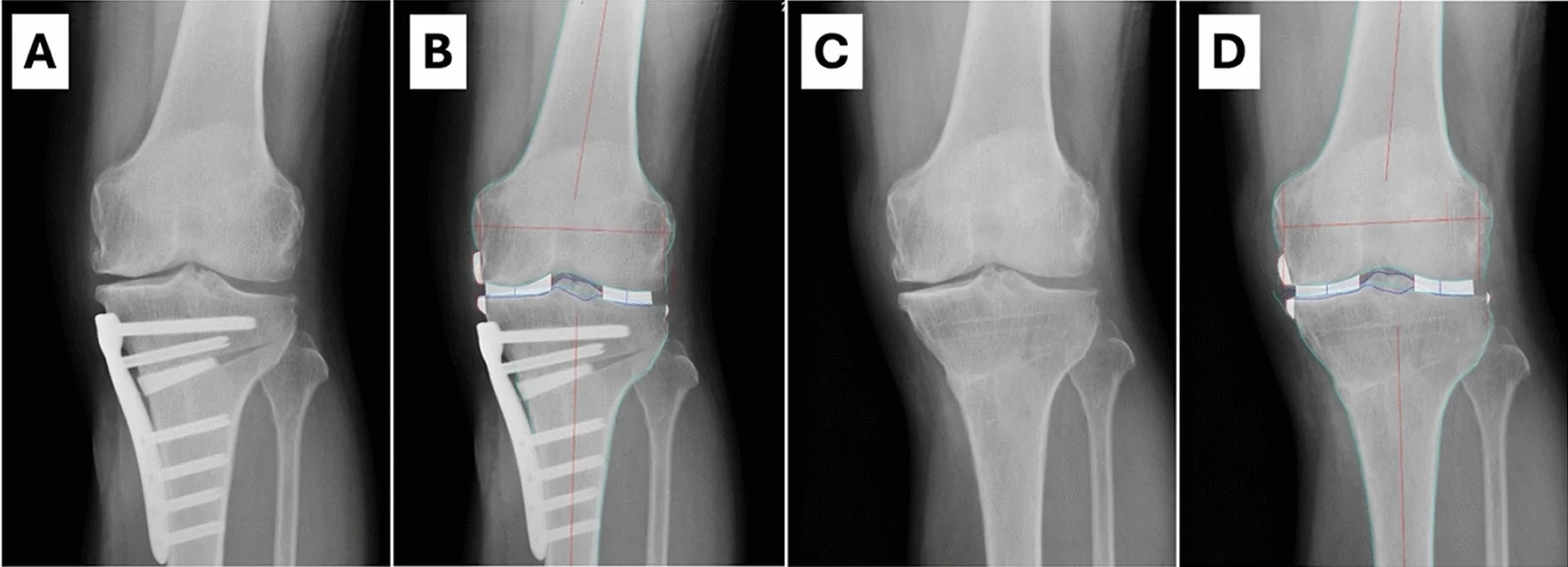
Quantitative evaluation of radiographic joint spaces and osteophytes using automatic measurement software. **A** Immediately after MOWHTO; **B**, **D** Quantitative measurements of joint spaces and osteophytes; **C** 3-year follow-up after MOWHTO. *MOWHTO* medial open-wedge high tibial osteotomy

Weight-bearing plain radiographs were acquired both until 1-month before and after the MOWHTO. We determined until 1-month after surgery as “postoperative period.” Additionally, plain radiographs were acquired 3 years after the MOWHTO to terminate the current follow-up (Fig. 1). All knee radiographic images were taken by experienced radiographic technicians with a film-focus distance of 130 cm, 50 kV, 200 mA, and 40 ms. During radiographic evaluation, both knees were in an orthostatic position and fully extended. According to the preoperative radiographs, knee osteoarthritis severity was determined using the Kellgren–Lawrence grade (KLG) [21]. After MOWHTO surgery, postoperative radiographs were installed in an automatic measurement software (KOACAD®, Inotech Co. Ltd, Hiroshima, Japan) with high reproducibility to measure JSW and osteophyte area (OPA) [22]. Identical radiographic acquisition protocols were applied at the postoperative baseline and 3-year follow-up assessments. Radiographic image quality and positioning were confirmed prior to KOACAD analysis to ensure measurement consistency. Medial and lateral JSW (MJSW and LJSW) were automatically measured at the narrowest joint-space point. Consistently, OPA was automatically measured in both the femur and tibia, and the summation of femoral and tibial OPA was calculated in each of the medial and lateral femorotibial compartments, respectively; these values were defined as medial and lateral OPA (MOPA and LOPA, Fig. 2) [22]. On the basis of the postoperative and 3-year follow-up radiographs, change in the amount of JSW and OPA was calculated using the following formula: ΔJSW/OPA = [JSW/OPA Three-year follow-up] − [JSW/OPA Postoperative period]. If patients demonstrate a ΔMJSW of ≤ −0.3 mm during a 3-year follow-up, we defined them as progressing medial knee OA (p-mOA) because the natural course of radiographic joint space narrowing in medial femorotibial compartment was reported as 0.1 mm per year [23]. By contrast, the patients demonstrated ΔMJSW > −0.3 mm during a 3-year follow-up; thus, we defined them as preventing medial knee OA (pr-mOA).

Regarding lower-limb alignment parameters, we measured the mechanical lateral distal femoral angle (mLDFA), MPTA, joint line convergence angle (JLCA), and %MA. Additionally, on the basis of preoperative and postoperative radiographs, the ΔMPTA, JLCA, and %MA were calculated using the following formula: ΔMPTA/JLCA/%MA = [Postoperative MPTA/JLCA/%MA] − [Preoperative MPTA/JLCA/%MA].

### Statistical analyses

Statistical analysis was performed using SPSS ver. 24.0 (SPSS Inc., Chicago, IL, USA). Distribution of all continuous values was analyzed using the Shapiro–Wilk test. According to the distribution of each continuous value, demographic data of radiographic parameters were compared using paired *t*-tests or Wilcoxon signed-rank test, and demographic data of categorical values were compared using the Chi-square or Fisher’s exact test. The Pearson’s or Spearman’s rank correlation coefficient was used to assess the correlation between preoperative and postoperative alignment parameters, amount of change in the alignment parameters, and amount of change in the radiographic knee OA parameters. Moreover, the optimal cut-off values for pMPTA and ΔMPTA to predict the prevalence of p-mOA were determined using receiver operating characteristic (ROC) curves. Finally, to control the interaction of bilateral knees, we performed a generalized estimating equations (GEE) with the prevalence of p-mOA as the dependent variable and with the cut-off of pMPTA or ΔMPTA as the independent variable, with adjustments for age, sex, BMI, the prevalence of medial meniscus meniscectomy, and medial joint space width at postoperative period. To validate the statistical significance of MPTA, sensitivity analyses were conducted by multiple linear regression model with ΔMJSW as the dependent variable and with pMPTA or ΔMPTA as the independent variable with adjustments for other alignment parameters such as postoperative %MA, Δ%MA, postoperative JLCA, and ΔJLCA. Statistical significance was defined as a value of *p* ≤ 0.05.

## Results

Mean opening width for the current MOWHTO surgery was 9.4 ± 2.1 mm (range: 5.0–15.0 mm). Preoperative, postoperative, and radiographic parameters are presented in Table 1. Mean preoperative MPTA was 84.6 ± 1.7°. After the current surgery, mean pMPTA was increased to 91.2 ± 2.2°; therefore, mean ΔMPTA was 6.6 ± 2.2° (Table 1). Categorical parameters of the patient demographics are also presented in Table 1. Of the 133 knees, 47 (35.3%) were defined as having p-mOA and 86 (64.7%) as pr-mOA. Preoperative KLG significantly worsened throughout the 3-year follow-up period in the overall population (mean value of the preoperative KLG was 1.6 ± 0.9, and that at 3 years of follow-up was 2.2 ± 0.8; *p* < 0.001 by Wilcoxon signed-rank test, Table 1). Additionally, the MJSW decreased from 3.8 ± 1.1 to 3.7 ± 1.1 mm. (*p* = 0.107, paired *t*-test). By contrast, LJSW significantly increased from 6.0 ± 1.0 to 6.2 ± 1.1 mm in the overall population (*p* = 0.001, paired *t*-test). The area of osteophytes significantly increased in both the medial and lateral compartments (MOPA: 23.3 ± 32.8 to 45.2 ± 36.5 mm^2^; LOPA: 6.8 ± 13.2 to 16.2 ± 19.9 mm^2^; *p* < 0.001, paired *t*-test). When the patient’s knees were divided into nonradiographic OA (ROA) with KLG 0–1 and ROA groups with KLG 2–4, the MJSW significantly decreased only in the non-ROA group (*p* = 0.004, paired *t*-test), and the LJSW significantly increased only in the ROA group (*p* = 0.003, paired *t*-test; Table 2).

**Table 1:** Demographic and radiographic data of the patients

Continuous values	Mean ± S.D.	Range
Age	57.5 ± 7.9	39 to 76
BMI (kg/m^2^)	26.3 ± 4.0	16.7 to 38.3
Opening width (mm)	9.4 ± 2.1	5.0 to 15.0
mLDFA	88.1 ± 2.0	82.8 to 93.1
MPTA_pre_	84.6 ± 1.7	78.7 to 88.0
MPTA_post_	91.2 ± 2.2	84.1 to 96.6
ΔMPTA	6.6 ± 2.2	1.7 to 13.1
JLCA_pre_	3.2 ± 1.4	0.9 to 7.4
JLCA_post_	2.7 ± 1.3	0.4 to 8.3
ΔJLCA	−0.5 ± 1.1	−4.9 to 3.5
%MA_pre_	20.8 ± 11.9	−14.7 to 52.2
%MA_post_	58.0 ± 10.7	22.8 to 86.8
Δ%MA	37.1 ± 13.2	−3.6 to 82.4

Categorical values	Number [%]
Unilateral/bilateral surgery	69 [68.3] / 32 [31.7]
Male/female patients	43 [42.6] / 58 [57.4]
Male/female knees	56 [42.1] / 77 [57.9]
MMX −/+	118 [88.7] / 15 [11.3]
MPTA < 90/≥ 90	37 [27.8] / 96 [72.2]
KLG preoperative period/3Y*	
0	15 [11.3] / 1 [0.8]
1	41 [30.8] / 18 [13.5]
2	57 [42.9] / 72 [54.1]
3	17 [12.8] / 36 [27.1]
4	3 [2.3] / 6 [4.5]
KLG progression −/+	71 [53.4] / 62 [46.6]
MOA progression −/+	104 [78.2] / 29 [21.8]

Statistical analysis: Fisher’s exact test (*p ≤ 0.05). Unilateral/Bilateral surgery and Male/Female are counted by total of 101 patients; the other categorical values are counted by total of 133 knees

*BMI* body mass index, *JLCA* joint line convergence angle, *JLCA*_*pre*_ preoperative JLCA, *JLCA*_*post*_ postoperative JLCA, *KLG* Kellgren–Lawrence grade, *mLDFA* mechanical lateral distal femoral angle, *MMX* medial meniscus meniscectomy, *MOA* medial knee osteoarthritis, *MPTA* medial proximal tibial angle, *MPTA*_*pre*_ preoperative MPTA, *MPTA*_*post*_ postperative MPTA, *%MA* % mechanical axis, *%MA*_*pre*_ preoperative %MA, *%MA*_*post*_ postoperative %MA, *SD* standard deviation, *3Y* 3-year follow-up

**Table 2:** Comparison of radiographic parameters between postoperative period and 3-year follow-up after medial open-wedge high tibial osteotomy

		Post Op	3 Year follow-up	Change of amount (Δ)
MJSW (mm)	Overall	3.8 ± 1.1	3.7 ± 1.1	−0.1 ± 0.8
	Non-ROA	4.5 ± 0.8	4.2 ± 1.0*	−0.3 ± 0.8
	ROA	3.3 ± 1.0	3.3 ± 1.1	0.0 ± 0.8
MOPA (mm^2^)	Overall	23.3 ± 32.8	45.2 ± 36.5*	21.9 ± 26.1
	Non-ROA	3.8 ± 6.4	21.8 ± 20.7*	18.0 ± 18.9
	ROA	37.6 ± 36.7	62.2 ± 36.1*	24.7 ± 30.1
LJSW (mm)	Overall	6.0 ± 1.0	6.2 ± 1.1*	0.2 ± 0.7
	Non-ROA	5.8 ± 1.0	5.9 ± 1.0	0.1 ± 0.7
	ROA	6.2 ± 1.0	6.5 ± 1.1*	0.3 ± 0.8
LOPA (mm^2^)	Overall	6.8 ± 13.2	16.2 ± 19.9*	9.5 ± 14.5
	Non-ROA	0.3 ± 2.5	5.2 ± 11.6*	4.8 ± 10.3
	ROA	11.4 ± 15.7	24.3 ± 20.8*	12.9 ± 16.2

Statistical analysis: paired *t*-tests (*p ≤ 0.05)

*LJSW* lateral joint space width, *LOPA* lateral osteophyte area, *MJSW* medial joint space width, *MOPA* medial osteophyte area, *Post Op* postoperative period (until 1 month after surgery), *ROA* radiographic knee osteoarthritis (Kellgren–Lawrence grade ≥ 2)

The correlations among preoperative, postoperative, and ΔMPTA parameters were demonstrated in Table 3. Overall, ΔMJSW positively correlated with postoperative MPTA and ΔMPTA. In the non-ROA group, ΔMJSW positively correlated with postoperative MPTA and ΔMPTA. These positive correlations were more robust than those observed in the overall population. However, there was no significant correlation between ΔMJSW and MPTA parameters in the ROA group. Additionally, no MPTA parameters correlated with ΔLJSW, ΔMOPA, and ΔLOPA (Table 3).

**Table 3:** Correlation between MPTA parameters and change in amount of radiographic parameters of knee osteoarthritis

	Overall				Non-ROA				ROA			
	ΔMJSW	ΔMOPA	ΔLJSW	ΔLOPA	ΔMJSW	ΔMOPA	ΔLJSW	ΔLOPA	ΔMJSW	ΔMOPA	ΔLJSW	ΔLOPA
MPTA_pre_	0.010	0.170	0.042	0.109	−0.008	0.158	0.078	0.080	0.019	0.186	0.013	0.132
MPTA_post_	0.298^*^	0.079	0.114	0.138	0.364*	−0.057	0.084	−0.111	0.167	0.077	0.083	0.110
ΔMPTA	0.289^*^	−0.049	0.083	0.056	0.393*	−0.208	0.017	−0.192	0.144	−0.062	0.069	0.009

Statistical analysis: Pearson’s correlation coefficient (*p ≤ 0.05)

*LJSW* lateral joint space width, *LOPA* lateral osteophyte area, *MJSW* medial joint space width, *MOPA* medial osteophyte area, *MPTA* medial proximal tibial angle, *MPTA*_*pre*_ preoperative MPTA, *MPTA*_*post*_ postoperative MPTA, *ROA* radiographic knee osteoarthritis (Kellgren–Lawrence grade ≥ 2)

**Fig. 3:**
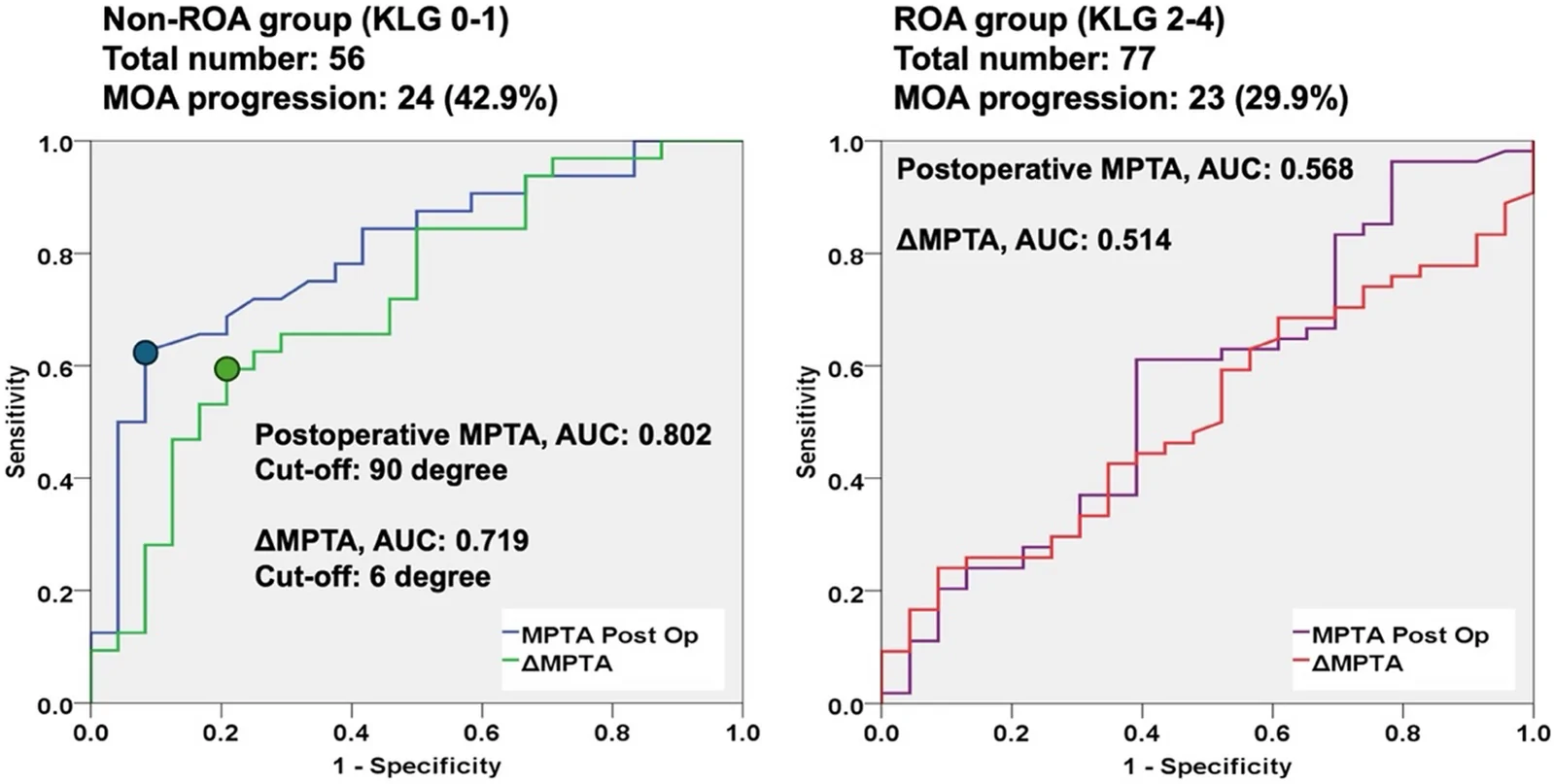
ROC curves to predict medial knee osteoarthritis progression (ΔMJSW ≤ −0.3 mm during a 3-year follow-up). *AUC* area under the curve, *MOA* medial knee osteoarthritis, *MPTA* medial proximal tibial angle, *ROA* radiographic knee osteoarthritis (Kellgren–Lawrence grade ≥ 2)

In particular, 24 (42.9%) of 56 knees in the non-ROA group demonstrated p-mOA; 23 (29.9%) of 77 knees in the ROA group demonstrated p-mOA. According to the current ROC curves, pMPTA [area under the curve (AUC): 0.802; 95% confidence interval (CI): 0.686–0.919] and ΔMPTA (AUC: 0.719; 95% CI: 0.582–0.855) could predict p-mOA only in the non-ROA group. The optimal cut-off for pMPTA was 90° and that of ΔMPTA was 6°. However, pMPTA and ΔMPTA did not predict p-mOA in the ROA group (Fig. 3). By contrast, neither pMPTA nor ΔMPTA was significantly associated with p-mOA in the ROA group (Table 4).

**Table 4:** Generalized estimating equations to evaluate the relationship among the prevalence of medial knee osteoarthritis progression, postoperative MPTA < 90°, and ΔMPTA 6°

	Group	Model	B	*p*-value	Odds ratio	95% CI
pMPTA 90°	Overall	Crude	0.334	0.001	1.396	1.149–1.700
		Adjusted	1.194	0.010	3.300	1.327–8.198
	Non-ROA	Crude	0.421	0.002	1.523	1.169–1.983
		Adjusted	1.688	0.014	5.408	1.400–20.905
	ROA	Crude	0.182	0.280	1.199	0.860–1.671
		Adjusted	0.777	0.343	2.174	0.436–10.837
ΔMPTA 6°	Overall	Crude	0.203	0.010	1.225	1.050–1.429
		Adjusted	0.785	0.031	2.192	1.072–4.481
	Non-ROA	Crude	0.385	0.001	1.469	1.169–1.844
		Adjusted	1.503	0.014	4.495	1.349–14.954
	ROA	Crude	0.008	0.942	1.008	0.810–1.249
		Adjusted	0.237	0.692	1.267	0.392–4.091

*B* regression coefficient, *pMPTA* postoperative medial proximal tibial angle, *ROA* radiographic knee osteoarthritis (Kellgren–Lawrence grade ≥ 2), *95% CI* 95% confidence interval

Dependent variable: prevalence of medial knee osteoarthritis progression; Independent variable: pMPTA 90° or ΔMPTA 6°; Adjusted variables: age, sex, body mass index, the prevalence of medial meniscus meniscectomy, and medial joint space width at postoperative period

On the basis of the optimal cut-off of 90-degree pMPTA to predict p-mOA, the current GEE demonstrated that patients with < 90° of pMPTA showed 3.3-times higher odds ratio of p-mOA compared with those with ≥ 90° of pMPTA (95% CI of odds ratio: 1.327–8.198; *B* = 1.194; *p* = 0.010) in overall; 5.408-times higher odds ratio of p-mOA compared with those with ≥ 90° of pMPTA (95% CI of odds ratio: 1.400–20.905; *B* = 1.688; *p* = 0.014) in non-ROA group. Consistently, patients with < 6° of ΔMPTA showed 2.192-times higher odds ratio of p-mOA compared with those with ≥ 6° of ΔMPTA (95% CI of odds ratio: 1.072–4.481; *B* = 0.785; *p* = 0.031) in overall; 4.495-times higher odds ratio of p-mOA compared with those with ≥ 6° of ΔMPTA (95% CI of odds ratio: 1.349–14.954; *B* = 1.503; *p* = 0.014, Table 4) in non-ROA group. Sensitivity analyses confirmed pMPTA was significantly correlated with ΔMJSW even after adjusting other alignment parameters such as postoperative %MA, Δ%MA, postoperative JLCA, and ΔJLCA. However, the correlation between ΔMPTA and ΔMJSW was attenuated after adjustment for other alignment parameters (Supplemental Table). Finally, Table 5 compared with the prevalence of p-mOA between < 90° and ≥ 90° of pMPTA.

**Table 5:** Comparison of the progression of medial knee osteoarthritis between ≤90° and ≥ 90° postoperative MPTA after medial open-wedge high tibial osteotomy

	Overall			Non-ROA group			ROA group		
	p-mOA			p-mOA			p-mOA		
	−	+	Total	−	+	Total	−	+	Total
pMPTA									
< 90°	15 (40.5%)	22 (59.5%)*	37	9 (34.6%)	17 (65.4%)*	26	6 (54.5%)	5 (45.5%)	11
≥ 90°	71 (74.0%)	25 (26.0%)	96	23 (76.7%)	7 (23.3%)	30	48 (72.7%)	18 (27.3%)	66
Total	86	47	133	32	24	56	54	23	77

Statistical Analysis: Chi-square or Fisher’s exact test (*p ≤ 0.05)

*pMPTA* postoperative medial proximal tibial angle, *p-mOA* progression of medial knee osteoarthritis, *ROA* radiographic knee osteoarthritis (Kellgren-Lawrence grade ≥ 2)

## Discussion

The primary finding of this study is that the odds ratio of the patients who demonstrated radiographic medial joint space narrowing (≤−0.3 mm/3 years) was significantly higher in those who underwent MOWHTO with pMPTA of < 90° or ΔMPTA of < 6°. By contrast, patients who underwent MOWHTO with pMPTA of ≥ 90° or ΔMPTA of ≥ 6° were less likely to demonstrate radiographic medial joint space narrowing. Therefore, throughout the 3-year follow-up period, patients whose pMPTA and ΔMPTA just after the MOWHTO had been sufficiently corrected by ≥ 90° or ≥ 6° could prevent their radiographic knee OA progression in the medial compartment. In particular, this primary finding was significantly robust in patients with early-stage radiographic knee OA such as those with KLG 0 or 1. Although the knee osteophytes enlarged during the 3-year follow-up, there was no significant correlation between ΔOPA and any of the MPTA parameters.

Limited evidence is available regarding the association between MPTA parameters and ΔMJSW. Consistent with our results, a previous longitudinal cohort study in which a 1° MPTA decrease significantly increased the odds of MJSW narrowing by 21% in medial knee OA, with a 2-year follow-up in populations with early-to-moderate OA (KLG 1, 2, or 3) [24]. In a more recent clinical study in patients who underwent lateral closing high tibial osteotomy, postoperative MJSW was significantly lower in a group with pMPTA correction of < 95° or ΔMPTA of < 8° compared with that in a group with pMPTA correction of ≥ 95° or ΔMPTA ≥ 8° [15]. By contrast, as for the conflicted data of this study, there was no significant difference in pMPTA (92.7° versus 91.9°) between patients with increased MJSW and decreased MJSW (0.8 mm versus −0.5 mm) at 3 years following MOWHTO [25]. This study supports the idea that a larger pMPTA can prevent the progression of medial joint space narrowing in patients who undergo MOWHTO. Notably, in accordance with the current data, the disease stage of medial knee OA is a significant factor in predicting the postoperative prognosis after MOWHTO. Although the actual mechanism remains unclear, if patients with medial compartment knee OA are in the early stage of radiographic knee OA, such as the KLG 0 or 1, MOWHTO would be more beneficial for them to prevent radiographic knee OA progression by correcting their pMPTA with ≥ 90°, compared with that in those having the advanced stage of radiographic knee OA.

Previous biomechanical studies have validated that a varus deformity consisting of a smaller MPTA is associated with higher mechanical loading on the medial compartment [2] or a higher knee adduction moment [26], which is associated with deterioration of the articular cartilage in the medial compartment [27–29]. Additionally, according to an MRI-based epidemiological study of healthy female participants, Ishibashi et al. reported that those with smaller MPTA demonstrated more severe bone marrow edema in their gonarthrotic subchondral bone [30], which also progresses to knee osteoarthritis [31, 32]. Therefore, if we are unable to correct the MPTA insufficiently during MOWHTO, there is a reduced chance of mitigating preoperative loading stress on the medial knee compartment, and the patients would miss the chance of preventing cartilage deterioration in the medial compartment, even after MOWHTO. During preoperative and intraoperative surgical planning, lowering pMPTA to a normal range (85–90°) can be challenging. In this context, the threshold of pMPTA 90° proposed in this study is expected to serve as a minimum necessary correction angle that prevents overcorrection while also helping to prevent progression of medial osteoarthritis.

At this point, debates related to the clinical interpretation of MJSW after HTO are still ongoing. Mostly, medial unicompartmental knee OA is characterized by a narrow MJSW [33] because sufficient data have validated that a narrow MJSW reflects the thickness of an articular cartilage in the medial femorotibial compartment [34] or the derangement of the medial meniscus [35]. Therefore, it is known that a decreased MJSW is proportional to the degree of arthritis corresponding to cartilage loss [25, 36–41]. Additionally, several previous studies have reported that increased MJSW is commonly related to the thickness and regeneration of articular cartilage after HTO [6–8, 33, 42]. However, it was also reported that MJSW enlargement after HTO was more related to lower limb alignment or mechanical conditions than to medial cartilage regeneration [43, 44]. According to an additional analysis based on post-operative weight-bearing line ratio (WBLR), no significant increase in MJSW was observed when the postoperative WBLR was < 60% [26]. Therefore, a change in the MJSW can be considered a radiographic parameter to assess whether the medial knee compartment has been effectively decompressed. In a more recent clinical study, the JLCA was identified as a factor affecting the MJSW after MOWHTO [36], and this is a factor that reflects soft tissue correction after HTO [18, 45–47]. Therefore, MCL release could be another contributor to the increased MJSW during MOWHTO [48, 49]. Because contact pressure is applied to the MCL with > 200 N when the correction angle increased by 10° during MOWHTO, it is inevitable for surgeons to release the MCL to reduce the applied pressure on the medial compartment during MOWHTO [2, 50, 51]. Although controversy still exists regarding the clinical interpretation of MJSW, increased MJSW is considered a factor that improves clinical outcomes after MOWHTO [18, 33, 44, 52, 53]. According to current and previous data, an increased MJSW by adequately increasing the MPTA during MOWHTO can alter the mechanical stress of the medial compartment and provide an anabolic environment for cartilage regeneration. Additionally, the disease stage of knee OA would affect the soft tissue contracture around the medial compartment, which alters the degree of ΔMJSW by increasing the MPTA during MOWHTO.

Even after we had performed MOWHTO, both pMPTA and ΔMPTA were not associated with ΔMOPA and ΔLOPA. The formation of osteophytes appears to be initiated from the neochondrogenesis of mesenchymal stem cells derived from the periosteum at the cartilage-bone junction [54] and the synovium [55]. The clearly visible osteophytes have consequently been developed through the accumulation of enchondral and intramembranous lamellar bone formation [56]. Several cytokines such as transforming growth factor-β [57] and bone morphogenetic proteins [58] are seen to promote the development of osteophytes. These chemical factors are regulated upstream via inflammatory agents such as the interleukin groups and MAP kinases. Although the cell source, histopathological survey, and chemical mediators concerned with osteophyte formation have been well studied, it still remains unclear how correcting lower limb alignment might control knee osteophyte formation. The current study may suggest that osteophyte progression in already osteoarthritic joints is driven predominantly by preexisting disease severity, synovial inflammation, or ongoing biological remodeling processes that are not fully modified by coronal-plane realignment alone within the relatively short-term follow-up period.

On the basis of the current 3-year follow-up data, there was no significant correlation between pMPTA and osteoarthritic changes, such as ΔLJSW and ΔLOPA, in the lateral compartment. Limited data are available on radiographic changes in the lateral compartment after MOWHTO, including osteophyte development. Excessive joint line obliquity due to an increased MPTA after HTO can lead to unfavorable knee kinematics and increased shear stress on the joint surface [2, 17, 59]. In more recent biomechanical studies, joint line obliquity of ≥ 5°, which approximately corresponds to an MPTA of 95°, induces detrimental stress on not only the articular cartilage of the lateral knee compartment [17], but also the lateral meniscus [38]. According to the actual clinical data of second look arthroscopy at approximately 1 year after MOWHTO, approximately 10–15% of patients demonstrated cartilage deterioration in the lateral femoral condyle and lateral tibial plateau [60, 61]. Additionally, the cartilage deterioration in the lateral compartment showed no significant differences between the increased (> 95°) and normal (≤95°) MPTAs [13, 60]. Regarding radiographic changes after MOWHTO, in 1987, Hernigou et al. reported that only 5 of 75 knees (6.7%) with valgus correction after MOWHTO showed lateral compartment osteoarthritis changes on plain radiographs, with a mean 11.5-year follow-up period [9]. Only a few studies have clarified the relationship between alterations in radiographic alignment and LJSW after MOWHTO. During an average of 20.9 ± 5.6 months after MOWHTO, Moon et al. reported that as postoperative WBLR increased, MJSW increased without significant changes in LJSW [44]. By contrast, Lee et al. reported serial changes in both MJSW and LJSW during a 2-year follow-up period after MOWHTO. They concluded that the LJSW decreased immediately after surgery, and this change was maintained for at least 2 years postoperatively. Moreover, the alteration in LJSW did not correlate with radiographic alignment parameters such as the hip-knee angle, mLDFA, MPTA, and JLCA [18]. On the basis of the current data, the LJSW significantly increased without narrowing of the MJSW during the 3-year follow-up period after MOWHTO. Consistently, ΔLJSW did not correlate with pMPTA and ΔMPTA. Although it is expected that overcorrection with greater pMPTA yields abnormal contact pressure of the lateral knee compartment owing to a shift in the MA [4, 20, 62], there is still controversy regarding the radiographic change in LJSW after MOWHTO in a short-term follow-up period. Further long-term observations are needed to determine how overcorrected pMPTA affects the derangement of the lateral compartment after MOWHTO including not only joint space narrowing, but also osteophyte formation.

This study has several limitations. First, we observed osteoarthritic changes using only plain radiograph with retrospective design; we could not conduct further detailed observations using MRI or arthroscopy. Second, the follow-up period in this study was relatively short (3 years), especially to evaluate lateral compartment deterioration. Third, the change in the patella–femur, which is also an important outcome during OWHTO, was not investigated [63–65]. Fourth, all the patients in this study underwent MOWHTO; therefore, the current results remain unclear for patients who underwent closed-wedge HTO. The postoperative knee biomechanics differ between open- and closed-wedge HTO in terms of altered patellar height, posterior tibial slope, and collateral laxity [66, 67]. Fifth, patients reported outcomes were not implemented in this study. Sixth, sample size in the current study was relatively underpowered to divide the subjects into two groups with non-ROA and ROA group; therefore, the 95% CIs of odds ratio in the current GEE models were more likely to be wide. Seventh, there is the potential of selection bias owing to the excluded patients. Eighth, the threshold of ΔMJSW ≤ −0.3 mm over 3 years is justified by citing a natural progression rate of 0.1 mm/year. A more validated threshold would be desirable for evaluating radiographic progression after HTO surgery. Finally, different surgeons and three different fixation devices (Puddu plate, Position HTO plate, and Tris Medial HTO plates) were involved in this study. These surgical heterogeneities would be confounders that could influence outcomes. Despite these several limitations, the current study is notable to provide the intraoperative reference of ≥ 90° pMPTA to prevent further radiographic progression of medial joint space narrowing after MOWHTO. Future studies will clarify the detailed mechanism of how pMPTA after MOWHTO prevents the progression of medial knee OA or affects the development of lateral knee OA.

## Conclusions

Only in the patients with KLG 0 or 1, the current longitudinal cohort study clarified that patients who underwent MOWHTO with pMPTA correction of < 90° demonstrated a higher odds ratio of significant medial joint space narrowing throughout the 3-year follow-up period, compared with those with pMPTA correction of ≥ 90°. Adequate correction with a cut-off of 90° intra- or post-operative MPTA is important when surgeons perform MOWHTO to prevent further radiographic progression of knee OA, especially in the patients without obvious osteoarthritic findings on the preoperative radiograph.

## Supplementary Information

Additional file 1.

## Data Availability

The current study’s data were not available unless the corresponding author permits to use.

## Abbreviations

ACL: Anterior cruciate ligament
AUC: Area under the curve
BMI: Body mass index
CI: Confidence interval
JLCA: Joint line convergence angle
JSW: Joint space width
KLG: Kellgren–Lawrence grade
LJSW: Lateral JSW
LOPA: Lateral OPA
MA: Mechanical axis
MCL: Medial collateral ligament
MJSW: Medial JSW
mLDFA: Mechanical lateral distal femoral angle
MOWHTO: Medial open-wedge high tibial osteotomy
MOPA: Medial OPA
MPTA: Medial proximal tibial angle
MRI: Magnetic resonance imaging
OA: Osteoarthritis
OPA: Osteophyte area
p-mOA: Progressing medial knee OA
pMPTA: Postoperative MPTA
pr-mOA: Preventing medial knee OA
ROA: Radiographic OA
ROC: Receiver operating characteristic
