# Supplementary material for: Adequately increased postoperative medial proximal tibial angle can prevent medial joint space narrowing after open-wedge high tibial osteotomy: a 3-year cohort study
Source: Knee Surg Relat Res. 2026 Oct 29;38:53. doi: 10.1186/s43019-026-00338-0 (PMC13721240; doi:10.1186/s43019-026-00338-0)
Supplement: Supplementary file 1 [file 43019_2026_338_MOESM1_ESM.docx]

**Supplemental Table: Sensitivity analyses to elucidate the correlation among ΔMJSW, postoperative MPTA, and ΔMPTA validated by additional alignment parameter**

|  |  | Additional variable | B, 95% CI | P value | VIF range | pMPTA (B, 95% CI) | P value | ΔMPTA (B, 95% CI) | P value |
| --- | --- | --- | --- | --- | --- | --- | --- | --- | --- |
| Non-ROA group | Model 1 | pJLCA | 0.136 (–0.071 to 0.343) | 0.192 | 1.08 to 1.18 | 0.108 (0.019 to 0.198) | 0.019 | 0.099 (–0.007 to 0.204) | 0.067 |
|  | Model 2 | ΔJLCA | 0.267 (0.060 to 0.474) | 0.013 | 1.12 to 1.34 | 0.127 (0.040 to 0.214) | 0.005 | 0.106 (0.003 to 0.209) | 0.044 |
|  | Model 3 | p%MA | <0.001 (–0.018 to 0.017) | 0.960 | 1.07 to 1.39 | 0.106 (0.008 to 0.205) | 0.035 | 0.093 (–0.025 to 0.211) | 0.120 |
|  | Model 4 | Δ%MA | 0.005 (–0.013 to 0.024) | 0.563 | 2.10 to 2.30 | 0.091 (–0.012 to 0.195) | 0.082 | 0.070 (–0.074 to 0.213) | 0.333 |
|  |  |  |  |  |  |  |  |  |  |
| ROA group | Model 1 | pJLCA | –0.112 (–0.266 to 0.043) | 0.153 | 1.16 to 1.36 | 0.071 (–0.027 to 0.169) | 0.154 | 0.093 (–0.003 to 0.189) | 0.056 |
|  | Model 2 | ΔJLCA | 0.042 (–0.113 to 0.197) | 0.589 | 1.07 to 1.20 | 0.061 (–0.037 to 0.159) | 0.218 | 0.060 (–0.032 to 0.152) | 0.197 |
|  | Model 3 | p%MA | 0.008 (–0.014 to 0.030) | 0.483 | 1.07 to 1.21 | 0.054 (–0.045 to 0.153) | 0.281 | 0.056 (–0.037 to 0.149) | 0.231 |
|  | Model 4 | Δ%MA | –0.004 (–0.025 to 0.016) | 0.683 | 1.94 to 2.24 | 0.057 (–0.048 to 0.161) | 0.282 | 0.078 (–0.043 to 0.200) | 0.204 |

Statistical analysis: multiple linear regression analysis. Independent variable: ΔMJSW; Dependent variable: postoperative MPTA or ΔMPTA; Adjusted variables: age, sex, body mass index, the prevalence of medial meniscus meniscectomy, medial joint space width at postoperative period, and additional alignment parameters.

B, regression coefficient; JLCA, joint line convergence angle; MPTA, medial proximal tibial angle; %MA, % mechanical axis; pJLCA: postoperative JLCA; pMPTA: postoperative MPTA; p%MA: postoperative %MA; ROA, radiographic knee osteoarthritis (Kellgren-Lawrence grade ≥2); 95% CI, 95% confidence interval.
